# Recent developments in quercetin nanomedicine and applications in osteoarthritis and rheumatoid arthritis therapies

**DOI:** 10.3389/fphar.2026.1790233

**Published:** 2026-02-27

**Authors:** Yuan Zhu, Hongxuan Chen, Yuanling Yi, Fuhui Zhou, Wenzhi Zhou, Shenghui Zhong

**Affiliations:** 1 Yichun University, Yichun, Jiangxi, China; 2 Xiamen Siming Meiyingyahan Medical Aesthetic Clinic, Xiamen, Fujian, China

**Keywords:** applications, nanoformulations, osteoarthritis, quercetin, rheumatoid arthritis

## Abstract

Quercetin has attracted increasing attention in the research of treatments for rheumatoid arthritis and osteoarthritis due to its excellent anti-inflammatory, antioxidant, and joint-protective effects. However, the bioavailability of quercetin is relatively low, primarily due to its poor solubility, rapid metabolism, and high clearance rate. Nanotechnology offers new opportunities to enhance the bioavailability of quercetin. Nanoformulations possess many advantages, such as a larger surface area, which can effectively improve the solubility of quercetin. Encapsulating quercetin in nanocarriers can prolong its residence time in the body, thereby improving its bioavailability and therapeutic efficacy. Targeted delivery can be achieved by modifying the surface of nanocarriers with ligands, allowing for direct transport of the drug to the site of injury and increasing the concentration at the lesion site. Nanocarriers can improve drug targeting and release behavior by adjusting their surface properties, thereby enhancing therapeutic effects. This article first summarizes the general preparation methods, drug-loading approaches, and common nanoformulations for quercetin. It then lists and summarizes the applications of quercetin nanoparticle formulations in rheumatoid arthritis and osteoarthritis. Finally, it concludes with a summary and outlook on the clinical applications and challenges associated with quercetin nanoformulations.

## 1 Introduction

Osteoarthritis (OA) and rheumatoid arthritis (RA), as common joint diseases, are chronic conditions that affect hundreds of millions of people worldwide. Their pathogenesis is complex and involves chronic inflammation, cell apoptosis, cartilaginous degeneration, and more (Ouyang et al., 2023). Although existing therapeutic drugs can alleviate symptoms, they often do not effectively control disease progression. Furthermore, long-term use of non-steroidal anti-inflammatory drugs (NSAIDs) and corticosteroids may lead to gastrointestinal complications and cardiovascular events (van Walsem et al., 2015; Dragos et al., 2017). Therefore, the search for new therapeutic agents with significant efficacy and minimal side effects has become a research hotspot.

Quercetin is a flavonoid compound widely present in plants, known for its excellent anti-inflammatory, antioxidant, and joint protective effects (Maurya and Maurya, 2022; Islam et al., 2023; Sul and Ra, 2021). Research indicates that quercetin can alleviate joint inflammation and related symptoms through various mechanisms, including inhibiting the release of inflammatory mediators, regulating immune responses, and promoting the proliferation of chondrocytes (Al-Khayri et al., 2022; Shen et al., 2021; Tang et al., 2022). Additionally, the protective effect of quercetin on articular cartilage is also well recognized. Despite its multiple bioactivities, quercetin has relatively low bioavailability in the body, mainly due to its poor water solubility, rapid metabolism, and high clearance rate. This makes it difficult for quercetin to reach optimal effective concentrations, thereby limiting its clinical applications. It has been reported that the bioavailability of quercetin is typically less than 20% (Manzoor et al., 2021; Georgiou et al., 2023).

The nanotechnology has provided a new opportunity to enhance the bioavailability of quercetin. Nanoparticle formulations have many advantages, such as a large surface area that can effectively enhance the solubility of quercetin. By encapsulating quercetin in nanocarriers, its residence time in the body can be prolonged, thereby improving its bioavailability and therapeutic effects (Zang et al., 2021; Joshi et al., 2023). Targeted delivery can be achieved by modifying ligands on the surface of nanocarriers, delivering drugs directly to the site of lesion and reducing damage to normal tissues. Nanocarriers can improve the targeting and release behavior of drugs by adjusting their surface properties, thus enhancing therapeutic effects The emergence of nanomedicine systems has made the clinical application of quercetin more feasible (Vishwas et al., 2023).

This review aims to systematically evaluate the potential of quercetin nanoformulations in the treatment of RA and OA. We summarized the loading capacity, release behavior, and therapeutic effects of different types of nanoformulations for quercetin in RA and OA models. By exploring the nanocarrier technologies of quercetin and their effects in applications, we hope to provide more effective solutions for their treatment. Through a review of existing literature, we aim to provide theoretical support and present experimental evidence for the application of quercetin nanoformulations in RA treatment. Additionally, we reveal the controversies and shortcomings in current research, which will guide future research directions.

## 2 Preparation of quercetin nanoformulations and encapsulation strategy

Quercetin is a polyphenolic flavonoid widely found in fruits, vegetables, and tea which is prone to oxidation and denaturation both *in vivo* and *in vitro*, leading to the loss of its pharmacological activity (Singh et al., 2023). Finding effective ways to maintain quercetin’s activity has become a crucial area of research regarding its nanoformulations and drug delivery methods. Identifying suitable methods for preparing quercetin nanoparticles and drug loading strategies is crucial for its eventual clinical application and poses the first obstacle that must be overcome (Xiong et al., 2020; Yadav et al., 2022). In this context, we summarize the commonly used preparation methods and drug delivery strategies for quercetin nanoformulations present in existing studies, comparing the advantages and disadvantages of the aforementioned methods.

### 2.1 Preparation of quercetin nanoformulations

The preparation methods of quercetin nanoparticle formulations mainly include the solvent evaporation method, ultrasonication-assisted method, self-assembly method, and spray drying method. The solvent evaporation method involves dissolving quercetin with polymers in organic solvents and then removing the solvent by evaporation to form nanoparticles. This method is simple and effective at controlling particle size (Mujtaba et al., 2019). Nonetheless, it has high demands in terms of large-scale production and solvent handling, which may have environmental impacts. The ultrasonication-assisted method utilizes the energy of ultrasound to thoroughly mix the drug and polymers, forming nanoparticles. In addition, the cavitation effect generated by ultrasound vibration, which serves to directly break down the quercetin dissolved in the organic solvent into nanoparticles in the liquid phase. This method can obtain uniform nanoparticles in a short period of time and has good adaptability for temperature-sensitive drugs (Jiang et al., 2022). The self-assembly method spontaneously forms nanostructures by changing environmental conditions such as pH and ionic strength, without the need for complex equipment and operations, although it has higher requirements for environmental conditions (Farr et al., 2018). The spray drying method involves spraying the solution of quercetin and a carrier into hot air to instantly dry and form nanoparticles. This method is suitable for large-scale production; however, it may lead to drug denaturation (Xu D. et al., 2023).

### 2.2 Encapsulation strategy

Quercetin is a naturally occurring flavonol compound characterized by a highly conjugated, rigid, and planar molecular skeleton, with the IUPAC name 3,3′,4′,5,7-pentahydroxyflavone. Its core structure consists of three ring systems: two benzene rings (Ring A and Ring B) connected via an oxygen-containing heterocyclic ring (Ring C, i.e., the pyranone ring), forming the classic C6–C3–C6 flavonoid backbone. This structure contains five phenolic hydroxyl groups (–OH) and one carbonyl group (C=O), which together serve as multiple hydrogen bond donors and acceptors. Meanwhile, the electron-rich π systems of Ring A and Ring B confer significant aromaticity and planarity, providing a structural basis for π–π stacking interactions. These structural features enable quercetin to be encapsulated by various carrier materials through multiple mechanisms. As shown in Table 1, the nanoformulations of quercetin reported in existing studies and their functional properties are presented, along with a summary of commonly used encapsulation methods.

**TABLE 1:** Common nanoformulations of quercetin for disease treatment.

Carrier	Preparation method	Formulation properties	Ref
Casein nanoparticles	Emulsion	Enhancement of bioavailability	Souza et al. (2021)
Carbon nanotubes	Impregnation and magnetic stirring	High surface area and thermal stability	Singh and Kumar (2022), Tang et al. (2021)
Chitosan Nanoliposomes	Facile electrostatic deposition	Enhancement of bioavailability	Ghosh et al. (2011)
Cross-linked chitosan Nanoliposomes	Ultrasonication	Enhancement of bioavailability	Hatahet et al. (2018)
Dietary fiber nanoparticles	Antisolvent precipitation	Improvement of solubility	Khor et al. (2017)
Liposomes	Thin film and high pressure homogeniser process	Improvement of solubility	Jonsson and Carlsten (2003), Wang et al. (2013), Lin et al. (2024)
Gold-quercetin conjugation nanoparticles	Solvent reduction method	Inhibits EGFR and capillary-like tube formation	Balakrishnan et al. (2017), Balakrishnan et al. (2016)
Metal–organic framework (ZIF-8) nanoparticles	Magnetic stirring	Enhanced drug accumulation and bioavailability	Sun et al. (2020), Hannan et al. (2023)
Metal oxide (Fe_3_O_4_) nanoparticles	Coprecipitation	High cellular uptake, magnetic resonance signal	Mashhadi Malekzadeh et al. (2017), Daglioglu (2017), Rezaei et al. (2019)
Metal oxide (ZnO) nanoparticles	Coprecipitation	High loading efficiency, pH-dependent release	Sadhukhan et al. (2019)
Mesoporous hydroxyapatite nanoparticles	Mesoporous template-assisted precipitation	Regulation of release rate	Joma et al. (2024)
PLGA nanoparticles	Solvent evaporation	Small particle size, high cellular uptake	Ersoz et al. (2020)
Polymeric micelles (PEG-PLGA)	Solvent-Water Phase separation	High entrapment efficiencies	Shen and TanTai (2020)
Polymeric micelles (Folate-PEG-PCL)	Solvent-Water phase separation	Mixed micelles	Khoee and Kavand (2014), Caddeo et al. (2017)
Silica nanoparticles	Oil-in-water microemulsion	Enhancement of bioavailability	Aghapour et al. (2018), Khattabi et al. (2023), Lee et al. (2016), Xu et al. (2019)
Other nanoparticles	Self-polymerization of quercetin	Self-polymerization of quercetin, high encapsulation and loading efficiency, enhancement in cellular uptake	Sunoqrot et al. (2018)

#### 2.2.1 Polymer encapsulation

Quercetin, as a natural active ingredient, exhibits various pharmacological effects such as anti-inflammatory, antioxidant, and antitumor activities. However, quercetin is prone to losing its efficacy due to external factors such as light, heat, and oxygen in in vitro conditions. Therefore, embedding quercetin in a polymer matrix has become an effective sustained-release technology, which can prolong its retention time in the body, enhance the duration of therapeutic effects, and make medication administration more convenient by avoiding frequent dosing, thus achieving better treatment outcomes (Das et al., 2024). This method not only protects quercetin from external environmental influences but also improves its stability, thereby enhancing its therapeutic effects (Yang et al., 2024).

#### 2.2.2 Physical adsorption

The method of immobilizing quercetin on the surfaces of nanocarriers through physical adsorption is a simple operational approach. However, studies have shown that drugs immobilized by this method release quickly *in vivo*, thereby making it difficult to maintain their efficacy over the long term (Wu et al., 2020). Therefore, further exploration of alternative carrier materials or modification methods is required to enhance drug stability and prolong the release time for improved therapeutic outcomes (Shen et al., 2022).

#### 2.2.3 Covalent bonding

Covalently bonding quercetin with carrier materials is an effective method to enhance its therapeutic efficacy. Quercetin contains multiple hydroxyl groups that can form ester bonds with carboxyl-containing carrier materials. The chemical bond connection allows for a stable association between quercetin and the carrier materials, resulting in a more stable drug delivery effect. This approach can not only prolong the retention time of quercetin in the body but also improve its targeting and bioavailability (Jiang et al., 2022; Zhou et al., 2022). However, despite the ability to enhance the controlled release of quercetin using this method, the synthesis process is relatively complex and requires strict conditions. Reactions must be carried out under specific temperature, pressure, and solvent conditions to ensure the efficiency and purity of the compound. Additionally, rigorous separation and purification processes are necessary to guarantee the quality of the product. Although the synthesis process is complicated, the method of covalently bonding quercetin with carrier materials can effectively enhance its therapeutic efficacy and reduce side effects, making it significant for the drug development of quercetin. Therefore, despite the strict synthesis conditions, this method remains a promising and worthwhile drug delivery strategy to explore and apply (Wan et al., 2021; Chen et al., 2022).

## 3 Quercetin nanofomulations for the treatment of arthritis

### 3.1 Quercetin nanofomulations for the treatment of rheumatoid arthritis

Applying nanotechnology to deliver quercetin as nanoparticles into the bodies of arthritis patients can enhance the bioavailability and therapeutic effect of the drug. With nanocarrier technology, researchers can reduce the metabolic rate of quercetin, thereby extending the duration of its efficacy (Oliveira et al., 2022). Research shows that this technology can significantly improve the stability and solubility of quercetin, thus enhancing its effectiveness (Vinayak and Maurya, 2019). Quercetin nanomedicine also possesses targeting properties, allowing for precise release of the drug at the lesion site. Moreover, due to the EVLIS(Enhanced Vascular Leakage and Inflammatory Stimulation) effect present in RA synovium (similar to the EPR effect in tumors), quercetin nanomedicine can accumulate passively. Furthermore, high expression of folate and CD44 receptors is found at the inflammatory site, which enables the modification of active targeting ligands on the surface of the nanocarrier, thus achieving targeted delivery of the drug to the site of arthritis inflammation (Nogueira et al., 2016; Cui et al., 2024; Siddique et al., 2022). In numerous practical preclinical studies, quercetin-loaded nanofomulations have achieved significant efficacy, resulting in notable relief of arthritis symptoms and pain in model animals with rheumatoid arthritis. Therefore, quercetin-loaded nano-drugs hold great promise for application in the treatment of rheumatoid arthritis and are expected to become one of the important therapeutic options for this disease in the future.

**FIGURE 1:**
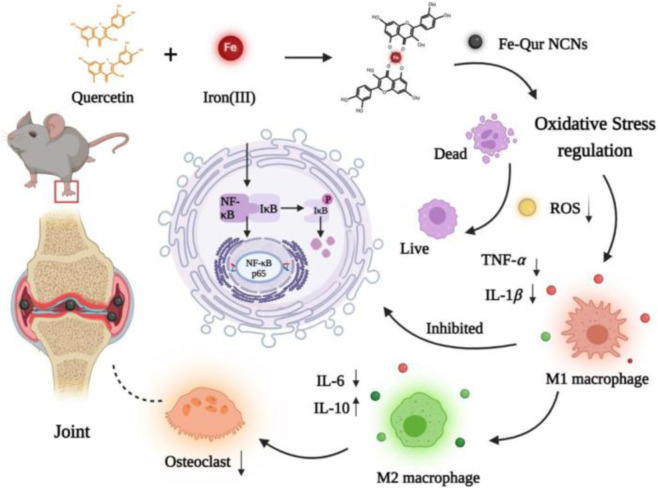
Ultrasmall iron‒quercetin metal natural product nanocomplex with antioxidant and macrophage regulation in rheumatoid arthritis. Reproduced from ref (Han et al., 2023). Copyright 2023 Elsevier.

Saleem et al. successfully prepared chitosan nanoparticles loaded with quercetin via a solvent evaporation method (Hannan et al., 2023). The hydrodynamic diameter of the nanoparticles is 83.9 nm, and the encapsulation efficiency of quercetin reaches 90.5%. In animal experiments, this nanoparticle formulation significantly alleviates joint swelling in FCA-induced RA model rats and reduces pro-inflammatory cytokine levels in plasma. Further histological examination of the joints reveals that the formulation could reduce the formation of synovial vascular hyperplasia and decrease bone damage. In addition, after treating the rats with a higher dose (20 mg/kg) of this formulation, there were no abnormalities observed in liver and kidney histology. This demonstrates that the nanoparticle formulation is virtually non-toxic and safe. In another study, Han et al. efficiently mixed Fe^3+^ with quercetin in a methanol solution to synthesize ultra-small Fe-Que nanoparticles (Han et al., 2023). These nanoparticles demonstrated excellent anti-inflammatory effects and the ability to downregulate ROS in both *in vivo* and *in vitro* experiments. Additionally, they can prevent macrophage polarization into pro-inflammatory macrophages by inhibiting the NF-κB pathway. Pharmacodynamic studies on RA model mice revealed that Fe-Que nanoparticles significantly alleviated joint swelling and inflammatory cell infiltration. Furthermore, they were able to prevent bone damage caused by bone resorption by increasing the number of anti-inflammatory macrophages in the joints (Figure 1).

**FIGURE 2:**
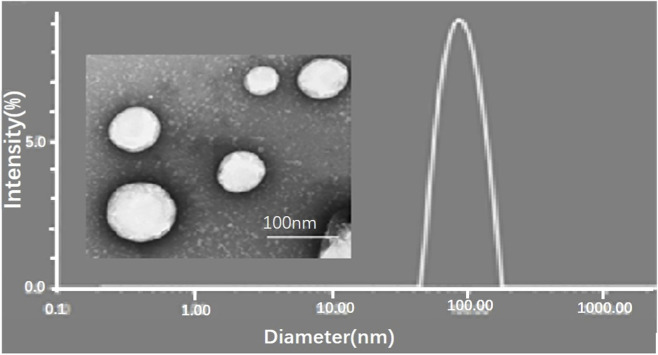
Transmission electron microscope (TEM) image and particle size distribution of PVGLIG-MTX-Que-Ms. Scale bar, 100 nm. Reproduced from ref (Li et al., 2024). Copyright 2024 Taylor and Francis.

Li et al. prepared microemulsions (PVGLIG-MTX-Que-Ms) through a film hydration method, with a hydrodynamic diameter of 89.6 nm (Figure 2) (Li et al., 2024). The PVGLIG in this system can be degraded by the highly expressed MMP-2 in the rheumatoid arthritis microenvironment, thus enabling the responsive release of Que at the joint site. Additionally, the surface-modified MTX can target the folate receptors on the membranes of activated macrophages. In in vitro release experiments, Que demonstrated sustained release characteristics. In cellular experiments, PVGLIG-MTX-Que-Ms exhibited low cytotoxicity and could be selectively taken up by inflammatory macrophages, significantly downregulating pro-inflammatory factors. Moreover, in the pharmacological study of CIA rats, it was found that this microemulsion could accumulate in inflamed joints, alleviating joint swelling and improving bone destruction. In drug safety evaluations, no significant toxicity of this system was observed. Table 2 summarizes the quercetin-loaded nanoformulations employed in existing rheumatoid arthritis treatment studies and their corresponding functional properties.

**TABLE 2:** Common nanoformulations of quercetin for RA treatment.

Carriers	Materials	Properties	Ref
Thioglycolic acid-capped cadmium telluride quantum dots nanoparticles	Cd2+,NaHTe, thioglycolic acid	Increased activities of antioxidant enzymes, reduce inflammation and improved cartilage regeneration in adjuvant induced arthritic Wistar rats	Jeyadevi et al. (2013)
Chitosan nanoparticles	Chitosan	Reduced TNF-α, IL-6 and ankle swelling in FCA induced arthritic rats	Hannan et al. (2023), Lawson (2023)
Solid lipid nanoparticles	Lipid	Reduced levels of inflammatory mediators such as tumor necrosis factor (TNF-α) and rheumatoid factor in FCA induced RA rat	Alotaibi et al. (2024)
Utralsmall iron-Quercetin nanoparticles	Fe^3+^, Quercetin	Largely reduced inflammatory cell infiltration, increased anti-inflammatory macrophage phenotypes, inhibit osteoclasts	Han et al. (2023)
Methotrexate modified quercetin micelles	DSPE-PEG2000-MTX	Exhibited slow-release properties, low cytotoxicity, strong targeting abilities, significantly reduced joint swelling, improved bone destruction	Li et al. (2024)
Nanoemulsion-based gel	oil phase- arachis oil and oleic acid (15%), surfactant tween 20 (6%) and co-surfactant PEG-400 (6%), Carbopol 940	Showed adequate rheological behavior with a good texture profile, improved drug permeation compared to free QCT gel, and inhibition of paw edema in rats induced by CFA over 24 h	Gokhale et al. (2019)
Microspheres	Polycaprolactone	Showed controlled release of quercetin in the joint cavity for more than 30 days by intra-articular injection to treat rheumatoid arthritis	Natarajan et al. (2011)

### 3.2 Quercetin nanofomulations for the treatment of osteoarthritis

Osteoarthritis (OA) is a common chronic joint disease, primarily characterized by degeneration of joint cartilage, bone proliferation, and synovitis, which severely affects patients’ quality of life (Abramoff and Caldera, 2020; Molnar et al., 2021). With the increasing aging population, the incidence of OA rises annually (Peat and Thomas, 2021; Hawker and King, 2022). Current treatment methods for osteoarthritis primarily include drug therapy, physical therapy, lifestyle interventions, and surgical options. Each of these methods has its own indications, and the choice is individualized based on the patient’s specific conditions (Jang et al., 2021). Drug therapy is currently the most common choice, usually involving NSAIDs, painkillers, and supplement-type medications such as glucosamine and chondroitin (van et al., 2020). These medications aim to relieve pain, reduce inflammation, and enhance function (Kolasinski et al., 2019; Katz et al., 2021). As previously mentioned, while NSAIDs and corticosteroids can provide therapeutic benefits in the anti-inflammatory treatment of OA, they are often associated with various side effects, particularly gastrointestinal damage (Migliorini et al., 2021; Yan et al., 2021). The use of NSAIDs frequently leads to adverse reactions in the gastrointestinal tract, including peptic ulcers and gastric bleeding. Studies have shown that long-term NSAID users have a significantly increased risk of gastrointestinal damage (Weng et al., 2023). This occurs because these medications inhibit the activity of cyclooxygenase (COX) enzymes, thereby reducing the synthesis of prostaglandins, which play a crucial role in protecting the gastric mucosa and maintaining normal gastrointestinal function. When prostaglandin levels decrease, the protective mechanisms of the gastric mucosa are compromised (Sohail et al., 2023; Bindu et al., 2020; Panchal and Prince Sabina, 2023).

There are numerous literature reports that quercetin has multifaceted therapeutic effects on OA. Studies have reported that quercetin alleviates joint inflammation by inhibiting the secretion of pro-inflammatory cytokines, such as tumor necrosis factor-alpha (TNF-α) and interleukin-1 beta (IL-1β) (Samadi et al., 2022; Wang H. et al., 2023). Additionally, quercetin can downregulate the activity of nuclear factor kappa B (NF-κB), thereby inhibiting inflammatory signaling pathways (Zhang et al., 2020; Shao et al., 2021; Wang and He, 2022). Oxidative stress is considered an important factor in the development of OA (Ansari et al., 2020; Riegger et al., 2023; Wang W. et al., 2023). As a potent antioxidant, quercetin can scavenge free radicals in the body, reducing oxidative damage. Quercetin protects chondrocytes from the effects of oxidative stress, thereby maintaining the structure and function of cartilage (Wang et al., 2022; Xi et al., 2023). Furthermore, research suggests that quercetin can promote the proliferation and differentiation of chondrocytes while upregulating the synthesis of cartilage matrix components, such as collagen and glycosaminoglycans. This protective and promoting effect on chondrocytes helps improve joint function and slow the progression of osteoarthritis. Additionally, animal experiments and some clinical studies have shown that quercetin has a significant effect in alleviating pain and improving joint mobility by regulating the biochemical environment within the joint cavity and reducing the sensation of pain (Lv et al., 2022; Mao et al., 2024).

Although quercetin has many advantages in the treatment of OA, as previously mentioned, its transition to clinical applications hinges on improving its solubility and bioavailability through formulation techniques due to its susceptibility to oxidative metabolism and poor water solubility. Therefore, nanotechnology naturally emerges as a promising solution to address this issue. Research on nano-drugs for treating OA primarily focuses on intra-articular injection formulations of nano-gels (Mok et al., 2020; Atwal et al., 2023; Xu Y. et al., 2023). Bahtiar et al. encapsulated quercetin in lecithin-chitosan nanoparticles and then prepared it into a gel (Permatasari et al., 2019). Subsequently, the results showed that this formulation significantly downregulated pro-inflammatory factors IL-1β and MMP-9, with MMP-9 playing an important role in the degradation of cartilage tissue during the onset and progression of OA. Histological analysis of rat knee joint tissue slices confirmed that the lecithin-chitosan nano-gel loaded with quercetin could indeed alleviate inflammation and improve cartilage integrity.

Kan and his team loaded quercetin into the metal-organic framework ZIF-8 to prepare Que@ZIF-8 nanoformulations for intra-articular injections (Kan et al., 2024). *In vitro* experiments showed that Que@ZIF-8 nanoparticles could release pH-responsive reagents into chondrocytes, effectively protecting them from interleukin (IL)-induced inflammation and apoptosis, thus promoting cartilage synthesis activity. The results suggested that the potential mechanism revealed a significant increase in autophagy in IL-β-treated chondrocytes, subsequently inhibiting the Pi3k/Akt signaling pathway, which plays a promoting role in the protective effect of Que@ZIF-8. In therapeutic experiments on unstable meniscal osteoarthritis (OA) mice, Que@ZIF-8 significantly improved the structural integrity of cartilage and chondrocyte status and slowed down the progression of OA. Importantly, due to its controlled release, Que@ZIF-8 demonstrated superiority over quercetin alone in the treatment of OA.

## 4 Discussion

Quercetin nanoformulations show promising clinical application prospects in the treatment of osteoarthritis and rheumatoid arthritis. By optimizing the design and preparation of nanoformulations, along with further clinical research, quercetin is expected to become a safe and effective treatment option. However, some challenges still need to be overcome, such as the standardization of production processes, the evaluation of long-term safety, and the implementation of large-scale clinical trials (Vishwas et al., 2023; Vieira et al., 2023). The use of organic solvents in the preparation of some nanomaterials raises concerns about solvent residues and contamination, which are key factors hindering their further application. Some nanocarrier materials may not be suitable for the treatment of RA and OA diseases. For instance, while Fe_3_O_4_ nanoparticles loaded with quercetin can be magnetically targeted to lesion sites, this material exhibits the Fenton effect, potentially inducing the production of more reactive oxygen species after accumulating in the joints, leading to an increase in inflammatory cells and pro-inflammatory factors that could negatively impact treatment outcomes (Ding et al., 2021; Gu et al., 2019). On the other hand, some carriers have supportive effects on arthritis treatment, such as calcium phosphate nanocarriers. Calcium phosphate, as a biocompatible material, possesses good osteoconductivity and bioactivity, promoting the proliferation and differentiation of chondrocytes or osteoblasts, which positively contributes to the regeneration and repair of articular cartilage (Zhang et al., 2022; Liu et al., 2022; Alam et al., 2017). Nanotargeted therapy for tumors has inspired researchers to design environment-responsive drug carriers tailored to the microenvironment of inflamed joints—characterized by low pH and abundant matrix metalloproteinases—yet this approach remains underexplored and warrants more extensive investigation. Overall, quercetin nanoformulations have potential in the treatment of osteoarthritis and rheumatoid arthritis, however, researchers need to take into account various factors such as biosafety, efficacy, and scalability in the design and construction of quercetin nanocarriers.
